# Alterations of Transcription of Genes Coding Anti-oxidative and Mitochondria-Related Proteins in Amyloid β Toxicity: Relevance to Alzheimer’s Disease

**DOI:** 10.1007/s12035-019-01819-y

**Published:** 2019-11-16

**Authors:** Magdalena Cieślik, Grzegorz A. Czapski, Sylwia Wójtowicz, Iga Wieczorek, Przemysław L. Wencel, Robert P. Strosznajder, Vivian Jaber, Walter J. Lukiw, Joanna B. Strosznajder

**Affiliations:** 1 Department of Cellular Signaling, Mossakowski Medical Research Centre Polish Academy of Sciences, Pawińskiego 5, 02-106 Warsaw, Poland; 2 Laboratory of Preclinical Research and Environmental Agents, Mossakowski Medical Research Centre Polish Academy of Sciences, Pawińskiego 5, 02-106 Warsaw, Poland; 3 LSU Neuroscience Center, Louisiana State University Health Sciences Center, New Orleans, LA 70112 USA; 4 Bollinger Professor of Alzheimer’s disease, LSU Neuroscience Center and Departments of Neurology and Ophthalmology, Louisiana State University Health Sciences Center, New Orleans, LA 70112 USA

**Keywords:** Neuronal cells, Microglial cells, Amyloid beta, Alzheimer’s disease, Gene expression, miRNA-146a

## Abstract

A growing body of evidence indicates that pathological forms of amyloid beta (Aβ) peptide contribute to neuronal degeneration and synaptic loss in Alzheimer’s disease (AD). In this study, we investigated the impact of exogenous Aβ_1-42_ oligomers (AβO) and endogenously liberated Aβ peptides on transcription of genes for anti-oxidative and mitochondria-related proteins in cell lines (neuronal SH-SY5Y and microglial BV2) and in brain cortex of transgenic AD (Tg-AD) mice, respectively. Our results demonstrated significant AβO-evoked changes in transcription of genes in SH-SY5Y cells, where AβO enhanced expression of *Sod1*, *Cat*, *mt-Nd1*, *Bcl2*, and attenuated *Sirt5*, *Sod2* and *Sdha*. In BV2 line, AβO increased the level of mRNA for *Sod2*, *Dnm1l*, *Bcl2*, and decreased for *Gpx4*, *Sirt1*, *Sirt3*, *mt-Nd1*, *Sdha* and *Mfn2*. Then, AβO enhanced free radicals level and impaired mitochondrial membrane potential only in SH-SY5Y cells, but reduced viability of both cell types. Inhibitor of poly(ADP-ribose)polymerase-1 and activator of sirtuin-1 more efficiently enhanced viability of SH-SY5Y than BV2 affected by AβO. Analysis of brain cortex of Tg-AD mice confirmed significant downregulation of *Sirt1, Mfn1* and *mt-Nd1* and upregulation of *Dnm1l*. In human AD brain, changes of microRNA pattern (miRNA-9, miRNA-34a, miRNA-146a and miRNA-155) seem to be responsible for decrease in *Sirt1* expression. Overall, our results demonstrated a diverse response of neuronal and microglial cells to AβO toxicity. Alterations of genes encoding Sirt1, Mfn1 and Drp1 in an experimental model of AD suggest that modulation of mitochondria dynamics and Sirt1, including miRNA strategy, may be crucial for improvement of AD therapy.

## Introduction

The amyloid beta (Aβ) cascade hypothesis of Alzheimer’s disease (AD), which was proposed originally by Hardy and Allsop [1], assumes that imbalance between production and clearance of Aβ in the brain leads to its accumulation, oligomerization, aggregation and formation of Aβ plaques. Aβ oligomers trigger a detrimental cascade leading inevitably to inflammatory neurodegeneration and dementia. More recent data demonstrated that small Aβ oligomers are the most toxic conformers of this peptide [2–4]. Correlation between level of Aβ oligomers and cognitive dysfunction in experimental studies supports amyloidogenic hypothesis of AD [5]. Oxidative stress and mitochondrial dysfunction play the most important role in the pathomechanism of AD. Several studies have also demonstrated the important role of the inflammatory processes in AD pathology [6–9].

In the brain, immune system is represented by microglial cells and the roles of these cells in the pathomechanism of AD are complex and not fully elucidated [10]. Activation of microglia, as a result of Aβ plaque deposition, leads to release of pro-inflammatory mediators and, in consequence, to further damaging brain tissue. On the other hand, microglia may internalize and degrade Aβ deposits, but this clearance capacity is lost in the presence of high levels of Aβ [11]. The recent studies extended our understanding of the involvement of microglia in AD pathology and identified detailed roles of microglia in synaptic stripping, neuronal loss and cognitive decline [12, 13].

Healthy and fit microglial cells seem to be a prerequisite for developing successful strategies for preventing and reducing Aβ peptide toxicity and inflammatory reactions in AD. The necessary condition of microglial well-being is the correct function of mitochondria. Deficiency of mitochondrial ATP may lead to apoptosis and declining microglia function in protecting surrounding neurons against Aβ toxicity [14]. Mitochondrial dysfunction, one of the earliest alterations of AD, is proposed to be crucial in brain aging and pathogenesis/progression of AD [15–18]. Mitochondria are the major source of reactive oxygen species (ROS) in the cell, but also the main target of ROS. Aβ accelerates ROS generation and possibly triggers a ‘vicious cycle’: ROS—mitochondrial impairment—ROS [19–21]. It is known that superoxide dismutase 2 (SOD2) and other enzymes, glutathione peroxidase (GPX), glutathione reductase (GR) and sirtuins (mammalian class III histone deacetylases), are engaged in anti-oxidative defence. Among the most important early molecular changes in AD is downregulation of Sirt1 and Sirt3. Also, mitochondria sirtuins (Sirt) play important roles in the regulation of transcription and activity of several anti-oxidative enzymes such as SOD2 and catalase [22, 23]. These both enzymes are implicated in metabolic control, mitochondria function and longevity by deacetylation of histones, transcription factors and other proteins, including anti-oxidative enzymes [23]. Sirt3 may directly deacetylate and stabilize 8-oxoguanidine-DNA glycosylase 1 (OGG1), a base repair mitochondrial and nuclear enzyme, promoting its capacity to repair mtDNA [23, 24]. Sirt1 plays important role in regulation of peroxisome proliferator-activated receptor-gamma coactivator1 alpha (PGC1-α), mitochondria biogenesis and mitophagy [25–28]. Moreover, Sirt1 is involved in regulation of amyloid precursor protein (APP) metabolism and Aβ level [26, 27]. Aβ-evoked decrease of Sirt1 and Sirt3 is responsible for upregulation of Tau level and acetylation [29, 30]. On the other hand, overexpression of Sirt1 significantly attenuated Aβ-evoked NF-κB signalling and protected against microglia-dependent neurodegeneration [31]. Moreover, neuroprotective and anti-inflammatory effects of resveratrol are likely related to its ability to activate sirtuins [32]. These enzymes are crucial in protection of mitochondria against dysfunction induced by Aβ [27, 33]. However, several studies supported ‘mitochondria cascade hypothesis for sporadic AD’ which underlined that primary mitochondria dysfunction is upstream of Aβ deposition and toxicity in pathogenesis of AD [16, 17]. The mechanisms by which Aβ oligomers affect mitochondria remain not entirely understood. It was recently demonstrated that Aβ evokes destabilization of mitochondrial proteome, mainly by impairment of pre-protein maturation [34]. Also, impairment of mitochondrial dynamics was demonstrated both in AD patients and in Aβ toxicity and oxidative stress [35–39]. Mitochondrial dynamics is regulated mainly by highly evolutionary conserved GTP-ases: dynamin-related protein 1 (Drp1), mitofusins 1 and 2 (Mfn1 and Mfn2) and optical atrophy 1 (Opa1) [40]. These all proteins participate in controlling assembly and stability of respiratory chain supercomplexes, in remodelling of mitochondrial structure and distribution of mitochondria through neuronal bodies, axons, dendrites and synapses [41–43]. Several molecular mechanisms are engaged in Drp1-dependent mitochondrial fragmentation. Recently, Manczak at al. [44] demonstrated the protective effects of reduced Drp1 against Aβ-induced mitochondria dysfunction and synaptic damage in murine AD model. Their findings suggest that partial reduction of Drp1 decreases Aβ production, mitochondria dysfunction, dynamics and also synaptic activity in Tg2576 AD mouse models.

The impact of oligomeric Aβ on expression of mitochondria-related genes in microglia has never been evaluated carefully. Global alterations of gene transcription in brain tissue may not reflect subtle differences in various cell populations in the brain. To overcome this problem, we analysed Aβ-evoked alterations of gene expression separately in two cell lines: microglial BV2 and neuronal SH-SY5Y. To verify *in vitro* data, we investigated also changes of gene expression in transgenic murine AD model and in human AD brain tissue.

## Material and Methods

### Chemicals

HFIP-treated amyloid β_1–42_ (Cat. No. A-1163-2) was obtained from rPeptide (rPeptide, Bogart, GA, USA). MitoScreen (JC-1) kit was from BD Biosciences (San Jose, CA, USA). Reagents for reverse transcription (High Capacity RNA-to-cDNA Master Mix) and quantitative PCR (Taqman Assays and Gene Expression Master Mix) were from Applied Biosystems (Foster City, CA, USA). Serum-free Neurobasal-A medium and supplement B27 were from Thermo Fisher Scientific Inc., MA USA. BD Protease inhibitor cocktail Complete was obtained from Roche Diagnostics GmbH (Mannheim, Germany). Olaparib, SRT1720, Dulbecco’s modified Eagle’s medium (DMEM), foetal bovine serum (FBS), horse serum (HS), penicillin, streptomycin, glutamine, 3-(4,5-dimethyl-2-tiazolilo)-2,5-diphenyl-2H-tetrazolium bromide (MTT), TRI-reagent, DNase I, DTT, collagen, anhydrous DMSO and all other reagents were obtained from Sigma-Aldrich (St. Louis, MO, USA).

### Cell Culture and Treatment

Murine microglial BV2 cells obtained as a gift from Prof. R. Donato (Department of Experimental Medicine and Biochemical Sciences, University of Perugia) and human neuroblastoma SH-SY5Y cells purchased from European Collection of Authenticated Cell Culture, Sigma-Aldrich (St. Louis, MO, USA), treated with Amyloid β oligomers (AβO) were used as *in vitro* model that recapitulates part of the AD pathology. The BV2 cells were cultured in RPMI supplemented with 5% heat-inactivated FBS, 2 mM L-glutamine, 50 U/ml penicillin, 50 μg/ml streptomycin in 5% CO_2_ atmosphere at 37 °C. The SH-SY5Y cells were cultured in F12/MEM medium supplemented with 15% heat-inactivated FBS, 1% non-essential amino acids, 50 U/ml penicillin, and 50 μg/ml streptomycin as well as L-glutamine in 5% CO_2_ atmosphere at 37 °C.

Amyloid β oligomerization was performed as described previously [45–47]. Additionally, Aβ_1-42_ with scrambled sequence (Aβscr, the same composition of amino acids but in random order), which was subjected to the same oligomerization protocol, was used as a negative control (data not shown). To avoid binding of Aβ by serum albumins, all experiments were performed in serum-free Neurobasal-A medium with B27 supplement. Equal BV2 and SH-SY5Y cell numbers were seeded into dishes or 96-well collagen-coated plates and after 24 h, they were treated for 24–48 h with freshly prepared oligomeric Aβ (1 μM, the concentration was chosen after the former analysis of the cell survival curve) or with specified compound administered 5 min before Aβ. Olaparib (3.3 μM), an inhibitor of PARP, and SRT1720 (0.1 μM), an activator SIRT, were dissolved in DMSO [48]. Appropriate solvent was added to respective controls.

### Mice Tg-AD Model

Female FVB-Tg(Thy1; APP LD2/B6) mice, aged 12 months, were used. The animals overexpressed human AβPP with the ‘London’ V717I mutation under control of a fragment of Thy1 promoter with specificity towards brain and spinal cord neurons (APP^+^). Mice that did not inherit the transgene were used as controls (APP^−^). Mice were bred under specific pathogen-free (SPF) conditions by the Animal House of the Mossakowski Medical Research Centre PAS, Warsaw, Poland. The mice were housed in controlled temperature and humidity conditions and 12-h light/dark cycle. The protocol was approved by the Warsaw Local Ethics Committee for Animal Experimentation. All applicable international, national and/or institutional guidelines for the care and use of animals were followed. All efforts were made to minimize suffering and to reduce the number of animals used. The experiments were performed in accordance with good laboratory practice protocols and quality assurance methods.

### Human Neocortical Tissue Samples

Post-mortem human neocortical tissues were handled in strict accordance with the ethics review board policies at donor institutions, and the Institutional Biosafety Committee/Institutional Review Board (IBC/IRB) Committee’s ethical guidelines (IBC#12323; IRB#6774) at the Louisiana State University Health Sciences Centre, School of Medicine, New Orleans LA 70112 USA.

### Determination of Cell Survival (MTT test)

Cellular viability and mitochondrial function were measured by the reduction of 3-(4,5-dimethyl-2-tiazolilo)-2,5-diphenyl-2H-tetrazolium bromide (MTT) to formazan as described previously [45].

### Measurement of the Level of Intracellular ROS

Measurement of the free radicals level was carried out using fluorescent indicator 2′,7′-dichlorodihydrofluorescein diacetate (H_2_DCF-DA) (Cayman Chemical Company), as described previously [46]. DCF fluorescence was measured using a microplate reader FLUOstar Omega (Ortenberg, Germany) at 485 nm ex./538 nm em.

### Determination of Mitochondrial Membrane Potential (ΔΨm)

Analysis of mitochondrial membrane potential (ΔΨm) was performed by using JC-1 BD MitoScreen (JC-1) kit according to the manufacturer’s instructions. The ratio red/green fluorescence is dependent on the mitochondrial membrane potential. Stained cells (10,000 per analysis) were examined on BD FACS Canto II flow cytometer. Cells incubated in the presence of protonophore and uncoupler of oxidative phosphorylation, carbonyl cyanide 3-chlorophenylhydrazone (CCCP), was used as positive control.

### Analysis of Gene Expression

RNA was isolated by using TRI reagent and purified by using DNase I according to the manufacturer’s instructions (Sigma-Aldrich). Reverse transcription was performed by using High Capacity cDNA Reverse Transcription Kit according to the manufacturer’s protocol (Applied Biosystems). The level of mRNA for studied genes was analysed by using TaqMan Gene Expression Assays (Applied Biosystems). *Bax* (Hs00150269_m1, Mm00432051_m1), *Bcl2* (Hs00608023_m1, Mm00477631_m1), *Cat* (Hs0056308_m1, Mm00437992_m1), *Dnm1l* (Hs01552605_m1, Mm01342903_m1), *Fis1* (Hs00211420_m1, Mm00481580_m1), *Gpx4* (Hs00989766_g1, Mm00515041_m1), *Mfn1* (Hs00966851_m1, Mm00612599_m1), *Mfn2* (Hs00208382_m1, Mm00500120_m1), *mt-Co1* (Hs02596864_m1, Mm04225243_g1), *mt-Cytb* (Hs02596867_s1, Mm04225271_g1), *mt-Nd1* (Hs02596873_s1, Mm04225274_s1), *Opa1* (Hs01047018_m1, Mm01349707_g1), *Sdha* (Hs00417200_m1, Mm01352366_m1), *Sirt1* (Hs01009005_m1, Mm00490762_m1), *Sirt3* (Hs00953477_m1, Mm00452131_m1), *Sirt4* (Hs00202033_m1, Mm01201915_m1), *Sirt5* (Hs00978335_m1, Mm01351576_m1), *Sod1* (Hs00533490_m1, Mm0134423_g1) and *Sod2* (Hs00167309_m1, Mm01313000_m1). After initial analysis of threshold cycle values (Ct), Actb (s99999903_m1, ACTB_4352341E) was used as a reference gene. Quantitative PCR was performed on an Applied Biosystems 7500 Real-Time PCR System using TaqMan Gene Expression Master Mix according to the manufacturer’s instructions. The relative levels of mRNA were calculated using the ΔΔCt method.

### RNA Purity Statement

The RNA used in these experiments was of exceptionally high quality; only short post-mortem interval (PMI) human brain tissues were used with PMIs of ~ 3 h or less. RNA quality was assessed using an Agilent 2100 Bioanalyzer (Agilent Technologies, Santa Clara CA, USA); the mean RNA integrity number (RIN) used in these experiments was between 8.05 and 8.1; all RNAs used had an RNA A260/280 of between 2.05 and 2.11; all RNA 28S/18S ratios ranged between 1.4 and 1.6, and the average RNA yield ranged between 1.2 and 1.5 total μg RNA/mg of wet weight of brain tissue. Importantly, there was no significant difference in RNA A260/280, RNA 28S/18S ratios or total RNA yield between the control and AD groups**.**

### miRNA Analysis of AD Brain Tissues, miRNA-mRNA Linking Assay and Bioinformatics Analysis

The microRNA (miRNA)-array analysis of three controls versus three AD brains; age- (mean control age 78.4 ± 5.7 years *N* = 3; mean AD age 79.3 ± 9.2 years *N* = 3), gender- (all female) and post-mortem interval (PMI)-matched controls and AD brain temporal lobes was performed. Isolation, quality control, purification, quantification and statistical analysis of brain-enriched miRNA-9, miRNA-34a, miRNA-146a, miRNA-155 and mRNA were undertaken as previously described [49, 50]. All human brain tissue analysis and miRNA analysis were performed as previously described [49, 50].

### Assessment of Enzymatic Activity of Complex IV of Mitochondrial Respiratory Chain

Activity of cytochrome c oxidase (COX) in cell lysate was measured according to Spinazzi et al. [51]. Cells (about 5 × 10^6^) were collected, washed three times with PBS, and cell pellet was flash-frozen in liquid nitrogen and stored at − 80 °C until analysis. To prepare lysate, pellet was suspended in 0.4 ml of 20 mM phosphate buffer (pH 7.5) and homogenized by passing through the needle using 1 ml syringe until homogeneous solution appeared. Then, the cell lysate was three times frozen and then thawed. Cytochrome c was reduced by incubation in the presence of 0.5 mM DTT for 20 min at room temperature in dark, the efficacy of cytochrome c reduction was checked by calculating the ratio of the absorbance 550 nm/565 nm (ratio > 6 indicates effective reduction). To measure cytochrome c oxidase activity, 40 μl of lysate was incubated with 25 μM reduced cytochrome c in 25 mM phosphate buffer pH 7.0 at room temperature. The decrease of absorbance at 550 nm was measured during 3 min. The extinction coefficient for reduced cytochrome c is 18.5 mM^−1^ × cm^−1^.

### Statistical Analysis

The results were expressed as mean values ± S.E.M. miRNA array data were analysed as previously described [50] using a two-way factorial analysis of variance (*p*, ANOVA) with programs and procedures in the SAS language (Statistical Analysis Institute, Cary, NC, USA). Other data were analysed using Student *t* test or one-way ANOVA with Bonferroni post hoc test among multiple groups by using GraphPad Prism version 5.0 (Graph Pad Software, San Diego, CA, USA). *P* values < 0.05 were considered significant.

## Results

### Alterations of ROS Formation, Mitochondrial Membrane Potential and Viability of BV2 and SH-SY5Y cells

The previous studies demonstrated the significant role of mitochondrial dysfunction in AD-related pathology. In our study, we used 1 μM Aβ which contains mostly monomers and small-size oligomeric assemblies, mainly trimers and tetramers. Characteristics of our preparation of Aβ oligomers (AβO) were published previously [45–47]. To identify cell type-specific responses, two cell lines were compared: neuronal-like SH-SY5Y and microglial BV2. To study the effect of AβO, both cell lines were treated in the same medium (Neurobasal A supplemented with B27) to exclude milieu-evoked differences.

**Fig. 1:**
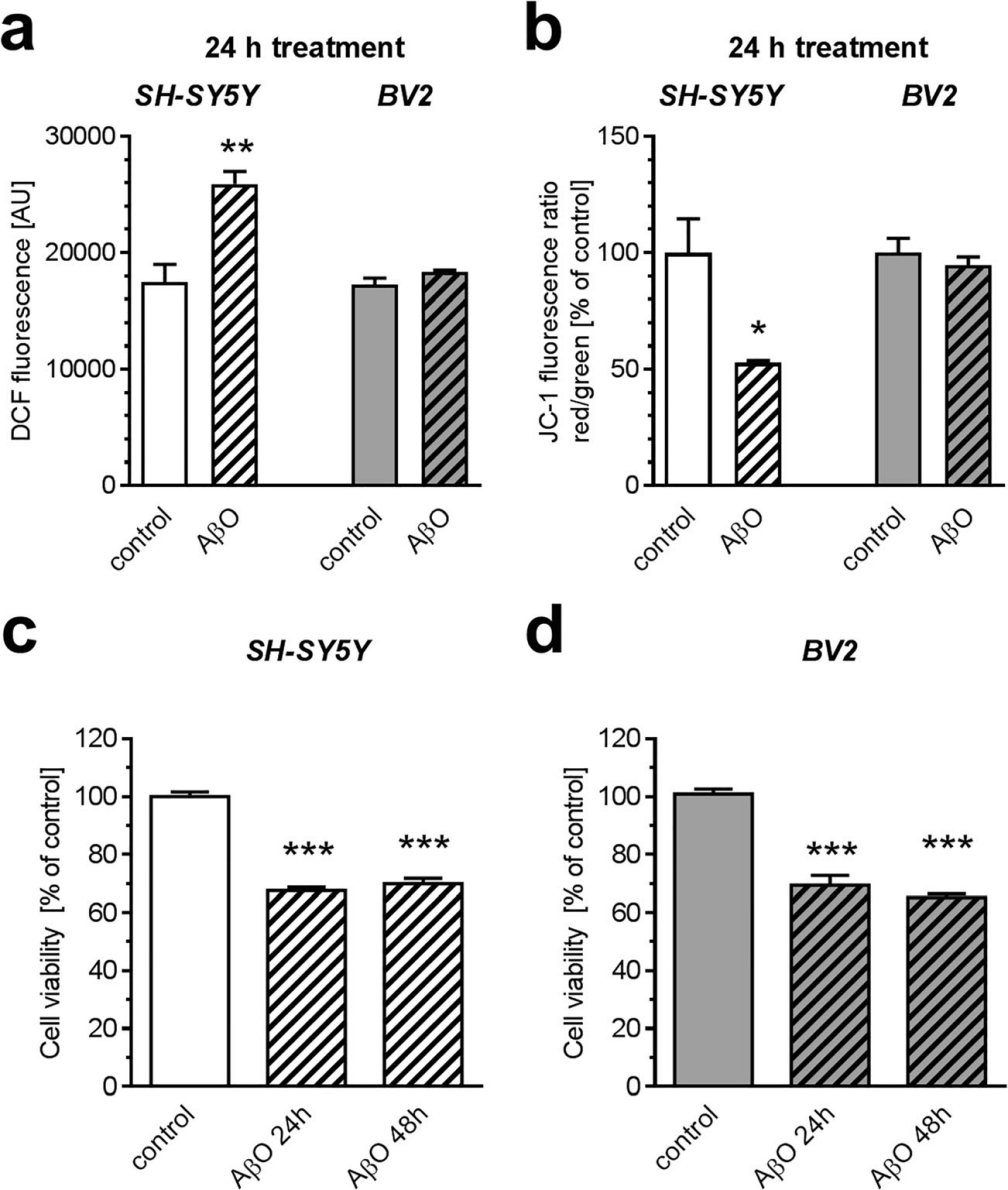
The effect of AβO on the level of reactive oxygen species, mitochondrial membrane potential (ΔΨm), and viability of SH-SY5Y and BV2 cells. SH-SY5Y and BV2 cells were incubated in the presence of oligomeric Aβ_1-42_ (AβO; 1 μM) for 24 h and 48 h. **a** The level of reactive oxygen species in cells was estimated by measuring fluorescence of DCF (*n* = 4). **b** ΔΨm was analyzed by using fluorescent dye JC-1 with flow-cytometric detection (*n* = 6). **c**, **d** Cell viability was measured by MTT assay after 24 and 48 h (*n* = 24). Data represent the mean value ± S.E.M. Statistical analysis was performed by using Student’s *t* test (**a**, **b**) or one-way ANOVA with Bonferroni post hoc test (**c**, **d**). *p < 0.05; ** *p* < 0.01; *** *p* < 0.001, as compared to the control group

As shown on Fig. 1a, after 24-h treatment with AβO, increased ROS formation was observed solely in SH-SY5Y (*p* = 0.008), but not in BV2 cells. Correspondingly, Aβ-evoked disruption of mitochondrial membrane potential was observed in SH-SY5Y cells (*p* = 0.0231), but not in BV2 cells (Fig. 1b). However, 24 h incubation in the presence of 1 μM AβO evoked significant decrease of cell viability, both in SH-SY5Y (*p* < 0.0001) and BV2 (*p* < 0.0001) cell line (Fig. 1c, d). Prolonged incubation (48 h) did not potentiate this effect.

### Alterations of Expression of Genes Related to Antioxidative Defence and Mitochondria in BV2 and SH-SY5Y cells

**Fig. 2:**
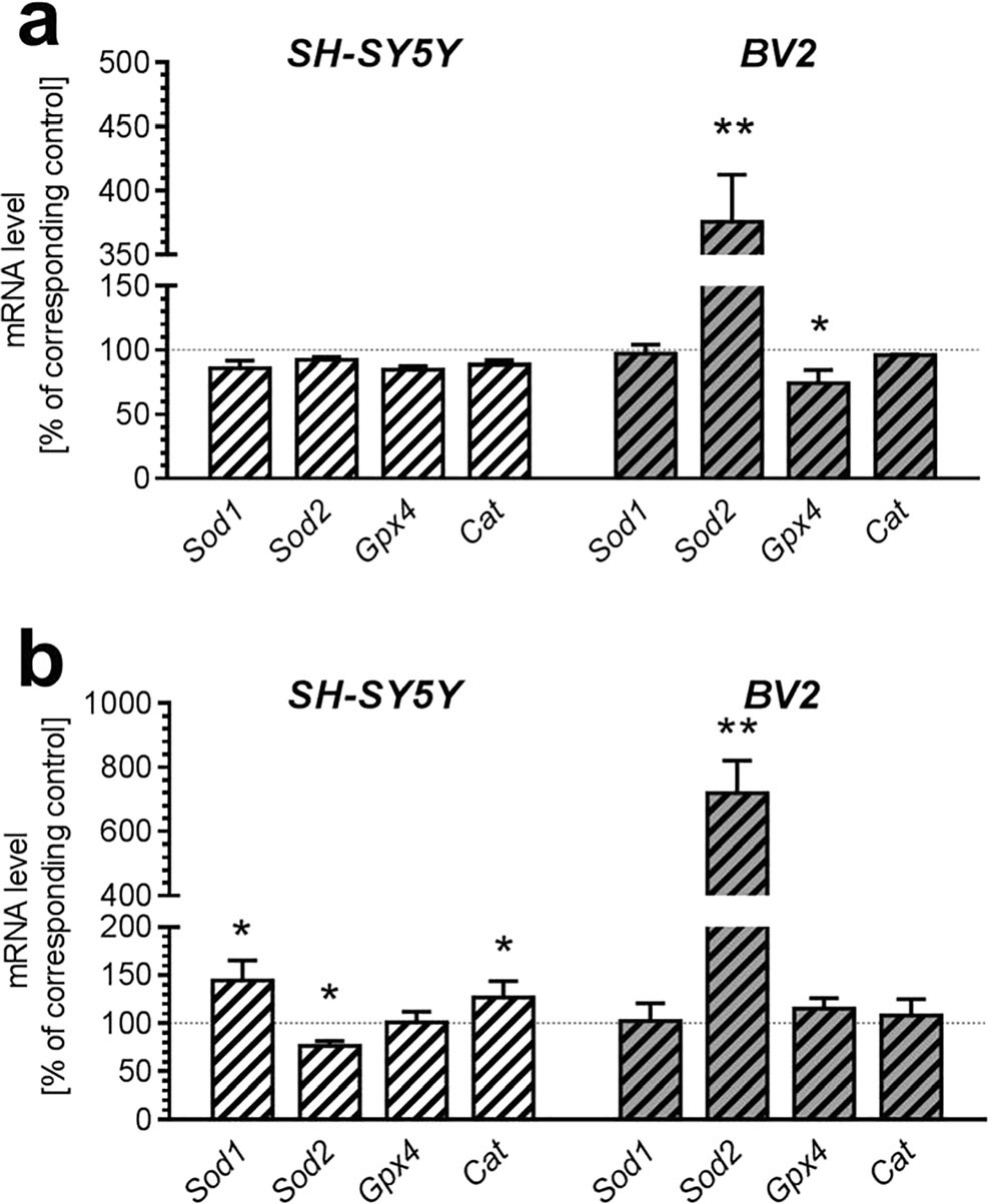
The effect of AβO on expression of genes for selected anti-oxidative enzymes in SH-SY5Y and BV2 cells. SH-SY5Y and BV2 cells were incubated in the presence of oligomeric Aβ_1-42_ (AβO; 1 μM) for 24 h (**a**) and 48 h (**b**). The levels of mRNA of superoxide dismutase 1 and 2 (*Sod1* and *Sod2*), glutathione peroxidase 4 (*Gpx4*) and catalase (*Cat*) were analyzed by quantitative RT-PCR. The results were normalized to *Actb* gene expression. Data represent the mean value ± S.E.M. for 5–8 independent experiments carried out in triplicate. Statistical analysis was performed by using Student’s *t* test. *p < 0.05; **p < 0.01, as compared to the respective control group

Consistently, transcription of proteins involved in antioxidative defence was different in SH-SY5Y and BV2 cells. After 24 h incubation, significant increase (up to 370% of control) in the level of mRNA for mitochondrial *Sod2* (*p* = 0.006) and decrease in the level of mRNA for *Gpx4* (*p* = 0.016) was observed exclusively in BV2 cells (Fig. 2a). Expression of other tested genes (*Sod1* and *Cat*) was not affected by 24 h incubation in the presence of AβO. After 48 h treatment, *Sod2* mRNA level raised to ca. 700% in BV2 cells (*p* = 0.0038), but in SH-SY5Y, it was slightly reduced (*p* = 0.0171) (Fig. 2b). On the contrary, at this time-point, transcription of *Sod1* was increased in SH-SY5Y cells (*p* = 0.0481), but not in BV2 cells. Similarly, mRNA level for *Cat* was also increased only in SH-SY5Y cells (*p* = 0.0366). Expression of gene for *Gpx4* was not affected by Aβ at this time-point.

**Fig. 3:**
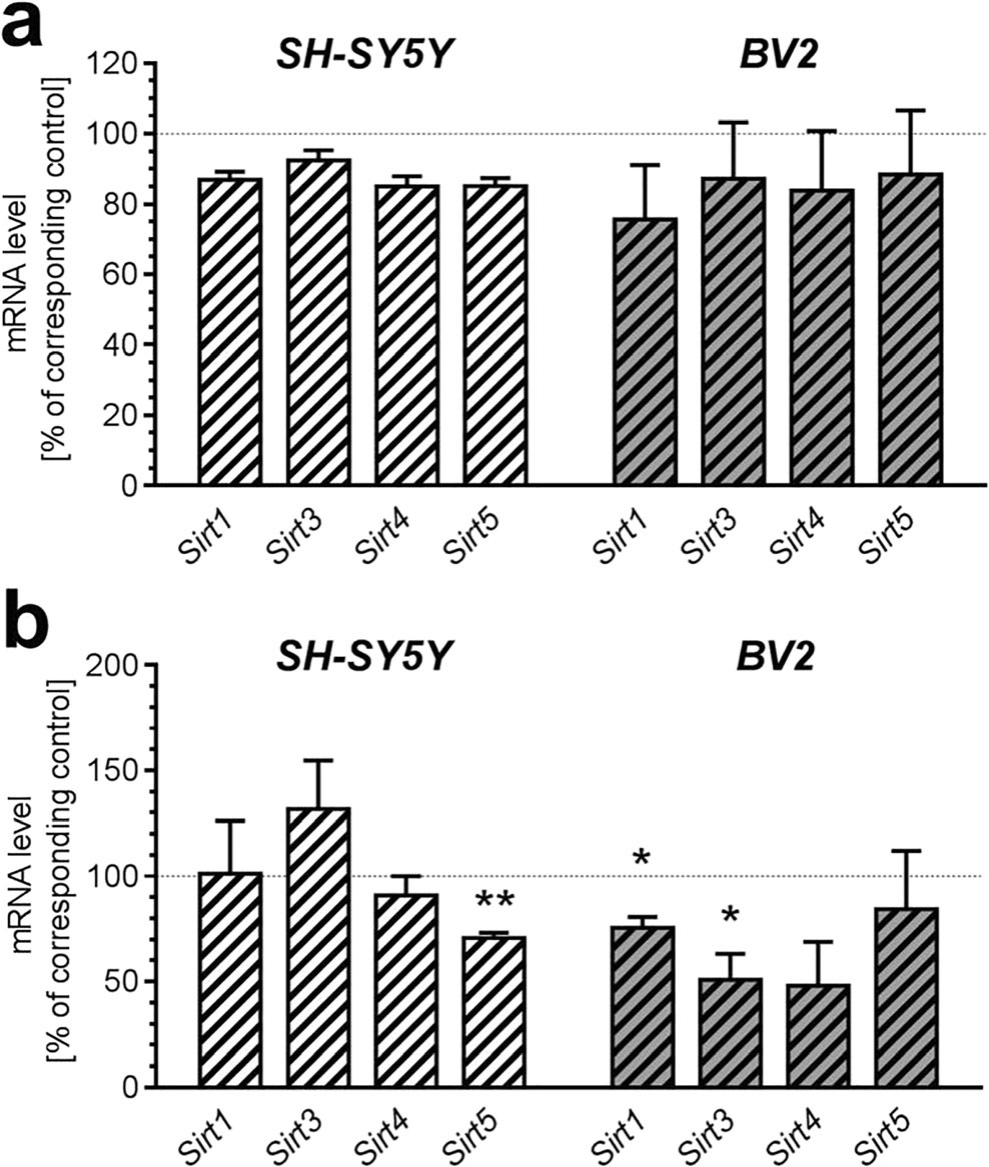
The effect of AβO on expression of genes for selected sirtuins in SH-SY5Y and BV2 cells. SH-SY5Y and BV2 cells were incubated in the presence of oligomeric Aβ_1-42_ (AβO; 1 μM) for 24 h (**a**) and 48 h (**b**). The levels of mRNA of *Sirt1* and mitochondrial sirtuins 3, 4, and 5 (*Sirt3*, *Sirt4*, *Sirt5*) were analyzed by quantitative RT-PCR. The results of RT-PCR were normalized to *Actb* gene expression. Data represent the mean value ± S.E.M. for 4–6 independent experiments carried out in triplicate. Statistical analysis was performed by using Student’s *t* test. *p < 0.05; **p < 0.01, as compared to the respective control group

Also, transcription of genes for *Sirt1* and mitochondrial sirtuins *Sirt3*, *-4*, *-5* contrasted in SH-SY5Y and BV2 cells (Fig. 3). Whereas 24 h incubation in the presence of AβO did not affect mRNA level of *Sirt1*, *Sirt3*, *Sirt4* and *Sirt5*, prolonged 48 h incubation significantly influenced expression of these genes differently in neuronal and microglial cells. AβO reduced expression of *Sirt1* (*p* = 0.0397) and *Sirt3* (*p* = 0.0341) in BV2, and decreased *Sirt5* (*p* = 0.003) in SH-SY5Y cells (Fig. 3b).

**Fig. 4:**
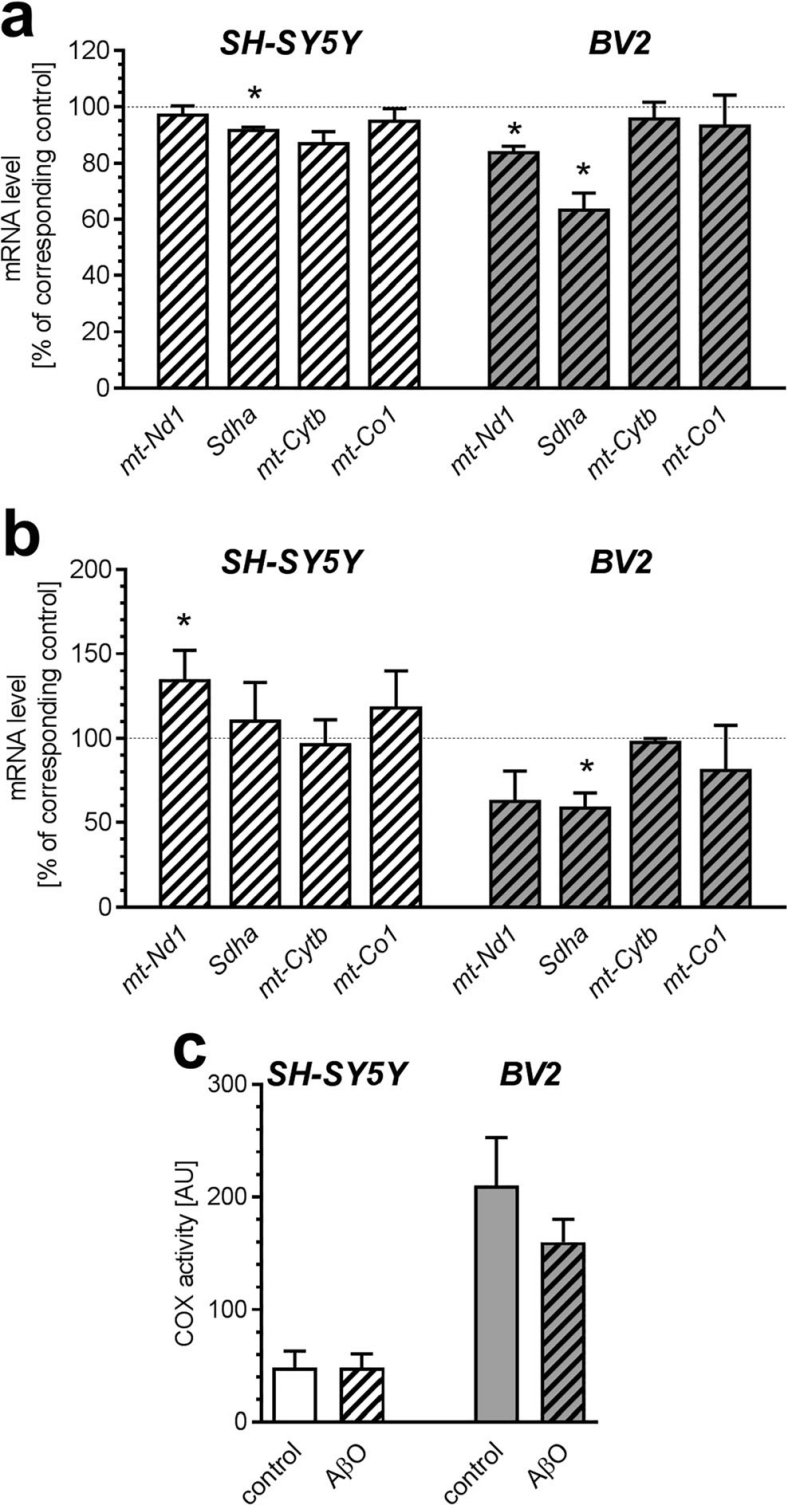
The effect of AβO on expression of genes for selected subunits of electron transport chain and cytochrome c oxidase activity in SH-SY5Y and BV2 cells. SH-SY5Y and BV2 cells were incubated in the presence of oligomeric Aβ_1-42_ (AβO; 1 μM) for 24 h (**a**) and 48 h (**b**). The levels of mRNA of mitochondrially encoded NADH dehydrogenase 1 (*mt-Nd1*), succinate dehydrogenase complex, subunit A flavoprotein (*Sdha*), mitochondrially encoded cytochrome b (*mt-Cytb*) and mitochondrially encoded cytochrome c oxidase I (*mt-Co1*) were analyzed by quantitative RT-PCR. The results of RT-PCR were normalized to *Actb* gene expression. **c** SH-SY5Y and BV2 cells were incubated in the presence of oligomeric Aβ_1-42_ (AβO; 1 μM) for 24 h. Activity of COX was estimated as described in “Materials and Methods”. Data represent the mean value ± S.E.M. for 4–6 independent experiments carried out in triplicate. Statistical analysis was performed by using Student’s *t* test. *p < 0.05, as compared to the respective control group

At the same time, a clear difference in expression of genes for mitochondrial electron transport chain (ETC) complexes was observed. After 24 h incubation, the level of mRNA for *Sdha* (*p* = 0.0345) and mitochondrially encoded *mt-Nd1* (*p* = 0.0423) was significantly decreased in BV2 line, but *mt-Cytb* and *mt-Co1* were not affected (Fig. 4a). Expression of *Sdha* (*p* = 0.0235) was slightly reduced in SH-SY5Y line, but other genes were not altered in this time-point. After prolonged 48 h incubation, reduced expression of *Sdha* in BV2 was maintained (*p* = 0.0154) but change in *mt-Nd1* was not statistically significant (*p* = 0.0556). Contrary, after 48 h treatment, mRNA level for *mt-Nd1* in SH-SY5Y was elevated (*p* = 0.0332) (Fig. 4b). Activity of cytochrome c oxidase (complex IV) was three times higher in BV2 cells comparing to SH-SY5Y (*p* = 0003); however, it was not affected by AβO (Fig. 4c).

**Fig. 5:**
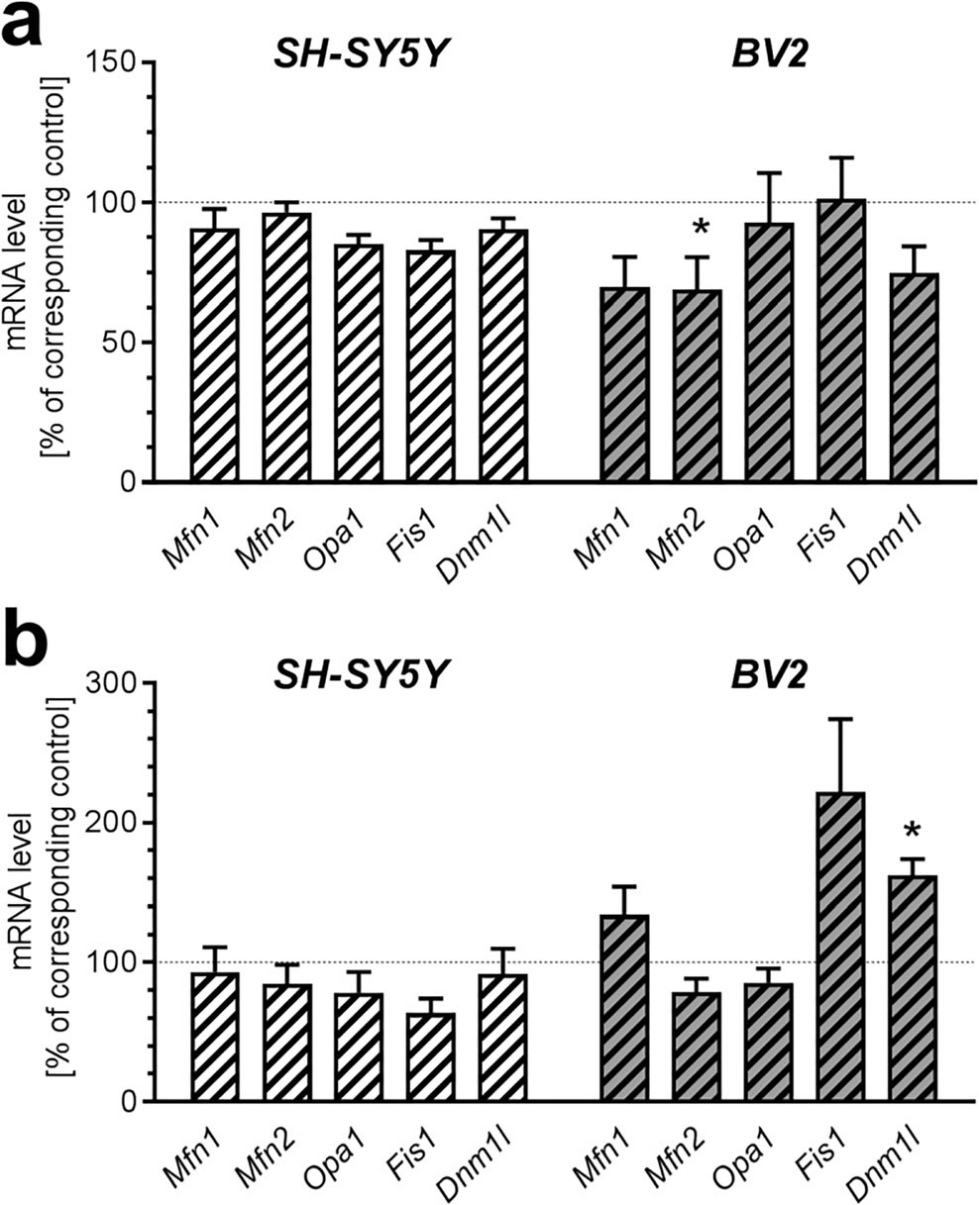
The effect of AβO on expression of genes for proteins involved in mitochondrial fission-fusion in SH-SY5Y and BV2 cells. SH-SY5Y and BV2 cells were incubated in the presence of oligomeric Aβ_1-42_ (AβO; 1 μM) for 24 h (**a**) and 48 h (**b**). The levels of mRNA of mitofusin 1 and 2 (*Mfn1* and *Mfn2*), optic atrophy 1 (*Opa1*), fission mitochondrial 1 (*Fis1*), dynamin 1-like, also known as DRP1 (*Dnm1l*) were analyzed by quantitative RT-PCR. The results of RT-PCR were normalized to *Actb* gene expression. Data represent the mean value ± S.E.M. for 5–6 independent experiments carried out in triplicate. Statistical analysis was performed by using Student’s *t* test. *p < 0.05, as compared to the respective control group

**Fig. 6:**
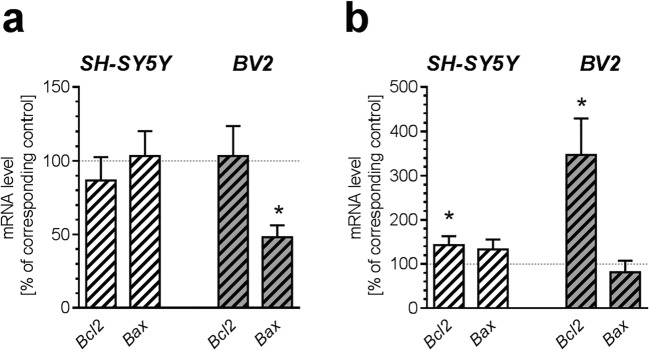
The effect of AβO on expression of genes for apoptosis-related proteins in SH-SY5Y and BV2 cells. SH-SY5Y and BV2 cells were incubated in the presence of oligomeric Aβ_1-42_ (AβO; 1 μM) for 24 h (**a**) and 48 h (**b**). The levels of mRNA for *Bcl2* and *Bax* were analyzed by quantitative RT-PCR. The results of RT-PCR were normalized to *Actb* gene expression. Data represent the mean value ± S.E.M. for 5–7 independent experiments carried out in triplicate. Statistical analysis was performed by using Student’s *t* test. * *p* < 0.05, as compared to the respective control group

Moreover, to analyse mitochondrial dynamics, expression of genes related to fusion-fission was measured. Incubation in the presence of AβO for 24 h reduced expression of *Mfn2* exclusively in BV2 cells (*p* = 0.029) but other tested genes were not affected (Fig. 5a). After 48 h incubation, AβO significantly enhanced mRNA level for *Dnm1l* (*p* = 0.0407) in BV2 cells (Fig. 5b). Similarly, to recognize cell type-dependent specific features of cell death mechanisms, we analysed the transcription of fundamental mitochondria-related supervisors of apoptosis—proapoptotic *Bax* and antiapoptotic *Bcl2*. Incubation in the presence of AβO for up to 24 h had no impact on mRNA level for *Bax* and *Bcl2* in SH-SY5Y line. However, in BV2 line, expression of the *Bax* gene was significantly reduced (*p* = 0.0242) and the mRNA level for *Bcl2* was not altered. In condition of prolonged 48 h incubation with AβO, expression of *Bcl2* was significantly increased in both cells lines (*p* = 0.0219 in SH-SY5Y and *p* = 0.0317 in BV2), but the increase in BV2 line seems to be more substantial. The mRNA level for *Bax* was not changed (Fig. 6).

### Effect of Pharmacological Modulation of NAD^+^-Dependent Enzymes on Viability of BV2 and SH-SY5Y Cells

**Fig. 7:**
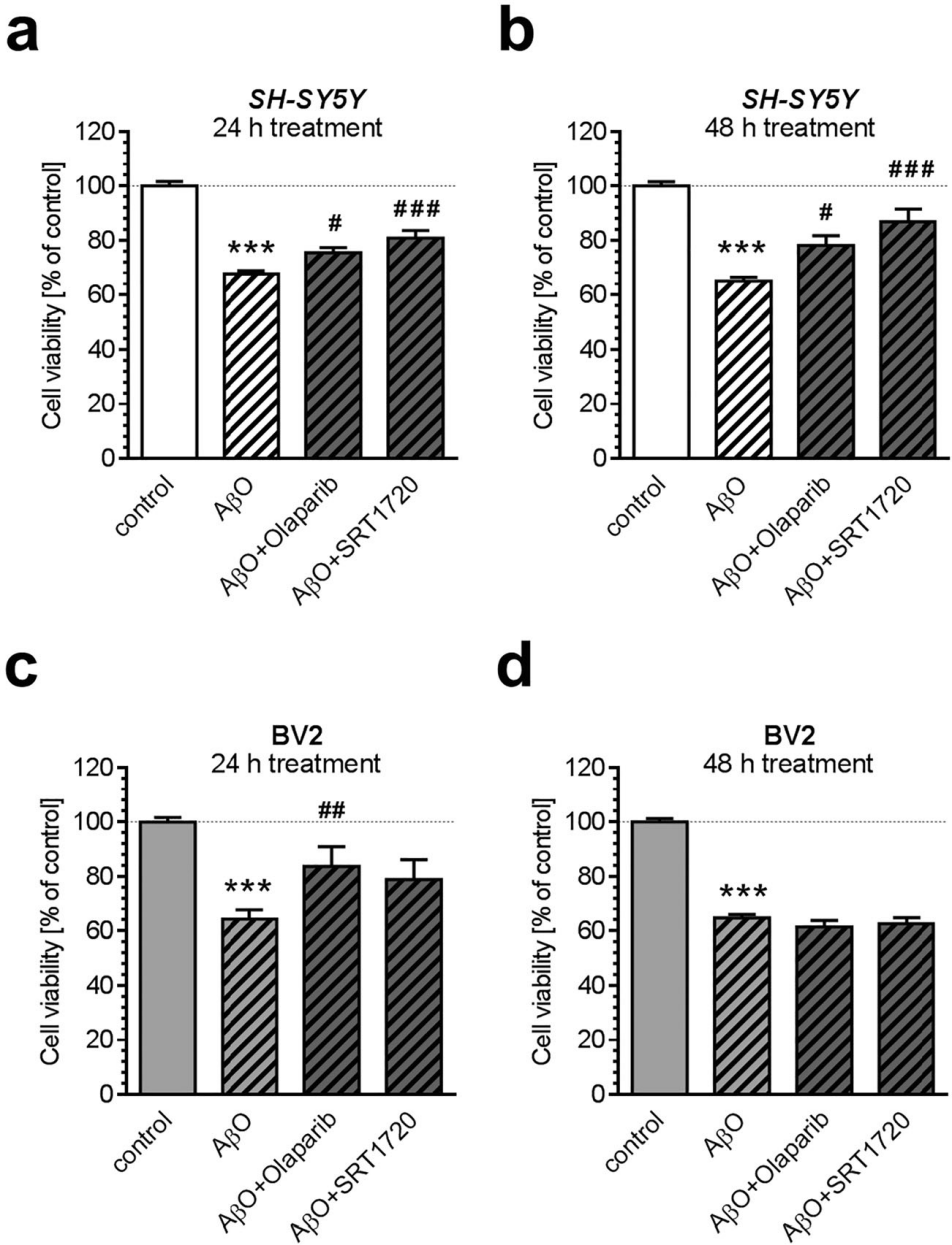
The effect of pharmacologically active compounds on AβO-evoked reduction of SH-SY5Y and BV2 cell viability. SH-SY5Y and BV2 cells were incubated for 24 (**a**, **c**) and 48 h (**b**, **d**) in the presence of oligomeric Aβ (AβO; 1 μM) and various pharmacologically active compounds: Olaparib (3.3 μM) and SRT1720 (0.1 μM). Cell viability was determined by using MTT assay, as described in “Materials and Methods”. Data represent the mean value ± S.E.M. for six (AβO + Olaparib and AβO + SRT1720) to 24 (AβO) independent experiments. ***p < 0.001, as compared to the control cells, #*p* < 0.05; ##*p* < 0.01; ###*p* < 0.001, as compared to AβO-treated group, by using a one-way ANOVA followed by Bonferroni post hoc test

In our study, we compared the role of NAD^+^-dependent enzymes, poly(ADP-ribose) polymerase-1 (PARP1) and sirtuins, in molecular mechanism of Aβ toxicity in SH-SY5Y and BV2 lines (Fig. 7). Co-incubation with PARP1 inhibitor, Olaparib, partially protected both SH-SY5Y (*p* = 0.0214 after 24 h and *p* = 0.0191 after 48 h) and BV2 (*p* = 0.0081 after 24 h) cells against AβO-evoked toxicity (*p* < 0.0001). Co-incubation with Sirt activator, SRT1720, reduced Aβ-triggered toxicity in SH-SY5Y (*p* < 0.0001 after 24 h and after 48 h), but had no effect in BV2 cells.

### Alterations of Expression of Genes for Proteins Related to Antioxidative Defence and Mitochondrial Function in Brain Cortex of Mouse Model of AD

**Fig. 8:**
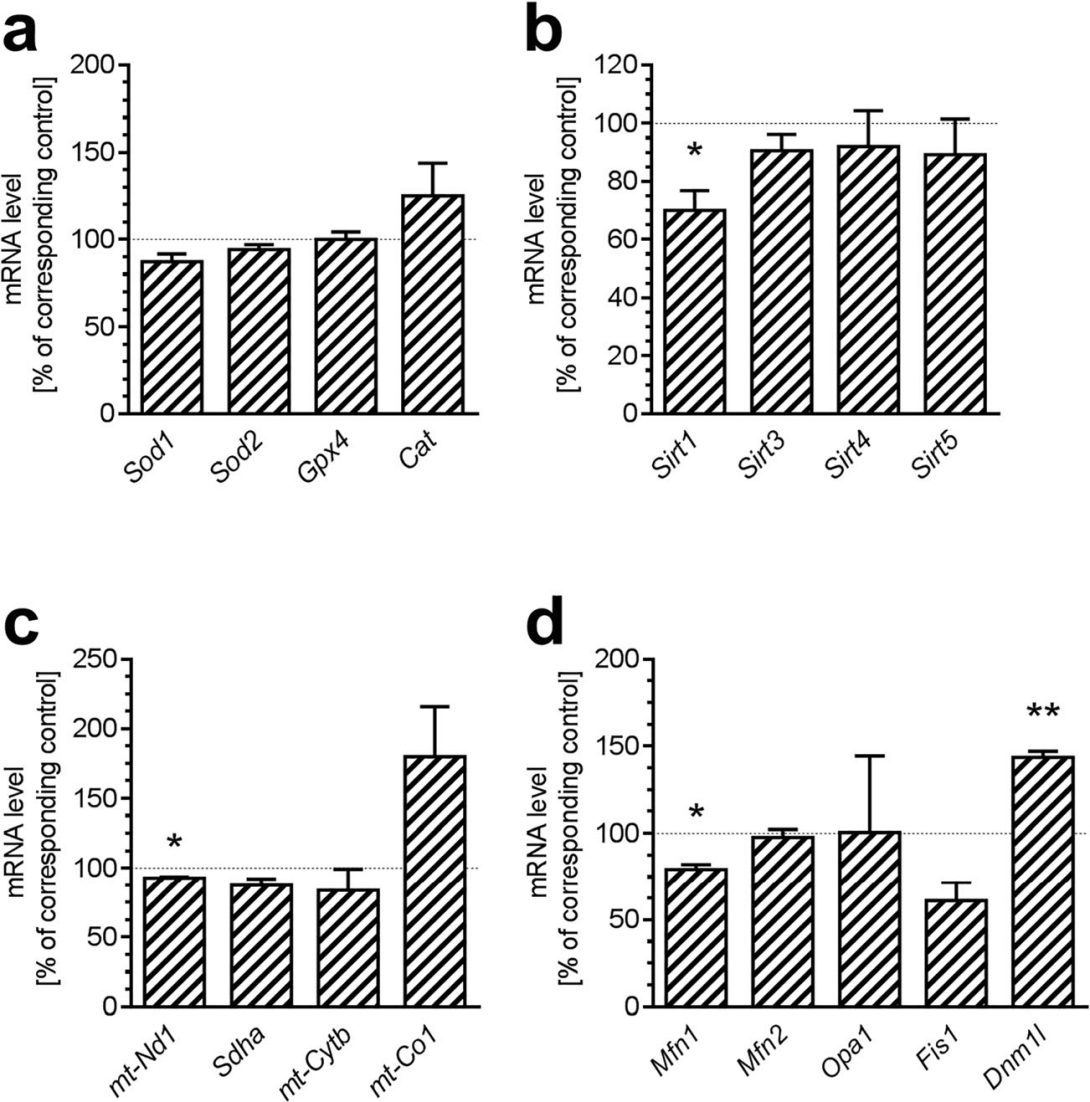
The effect of expression of human *APP* gene in mice on mRNA levels for mitochondria-related genes in brain cortex. Expression of genes in brain cortex of 12 month old male mice, APP^−^ and APP^+^ was analysed by quantitative RT-PCR. The results of RT-PCR were normalized to *Actb* gene expression. Data represent the mean value ± S.E.M. for three animals in each group. Statistical analysis was performed by using Student’s *t* test. *p < 0.05; **p < 0.01, as compared to the respective control group

To assess whether analogous changes in gene expression induced by Aβ occur *in vivo*, we analysed mRNA levels in animal model of Alzheimer’s disease. As shown in Fig. 8, in 12-month-old APP^+^ mice, which stably overexpress human *APP* gene with London mutation, mRNA levels for several mitochondria-related genes in brain cortex are altered, comparing to control animals. As shown in Fig. 8a, the levels of mRNA for antioxidative enzymes (*Sod1*, *Sod2*, *Gpx4*, *Cat*) were not changed in APP^+^ mice, comparing to control APP^-.^. Among tested sirtuins, only expression of *Sirt1* was reduced (Fig. 8b; *p* = 0.0383); expression of other sirtuins was not affected. Analysis of mRNA for complexes of mitochondrial electron transport chain (Fig. 8c) revealed slight decrease in the level of mRNA for mitochondrially encoded *mt-Nd1* (*p* = 0.0138), but changes of other tested genes were not statistically significant. The last group tested in mouse model of AD were genes for proteins involved in mitochondrial dynamics (Fig. 8d). It was found that mRNA level for *Mfn1* that is responsible for mitochondrial fusion was reduced (*p* = 0.0111), whereas expression of *Dnm1l* that regulates mitochondrial fission was significantly increased (*p* = 0.0019). The level of mRNA for other tested genes was not changed.

### Alterations of Expression of miRNA Related to Post-Transcriptional Regulating of Sirt1 Gene in Brain Cortex of AD Patients

**Fig. 9:**
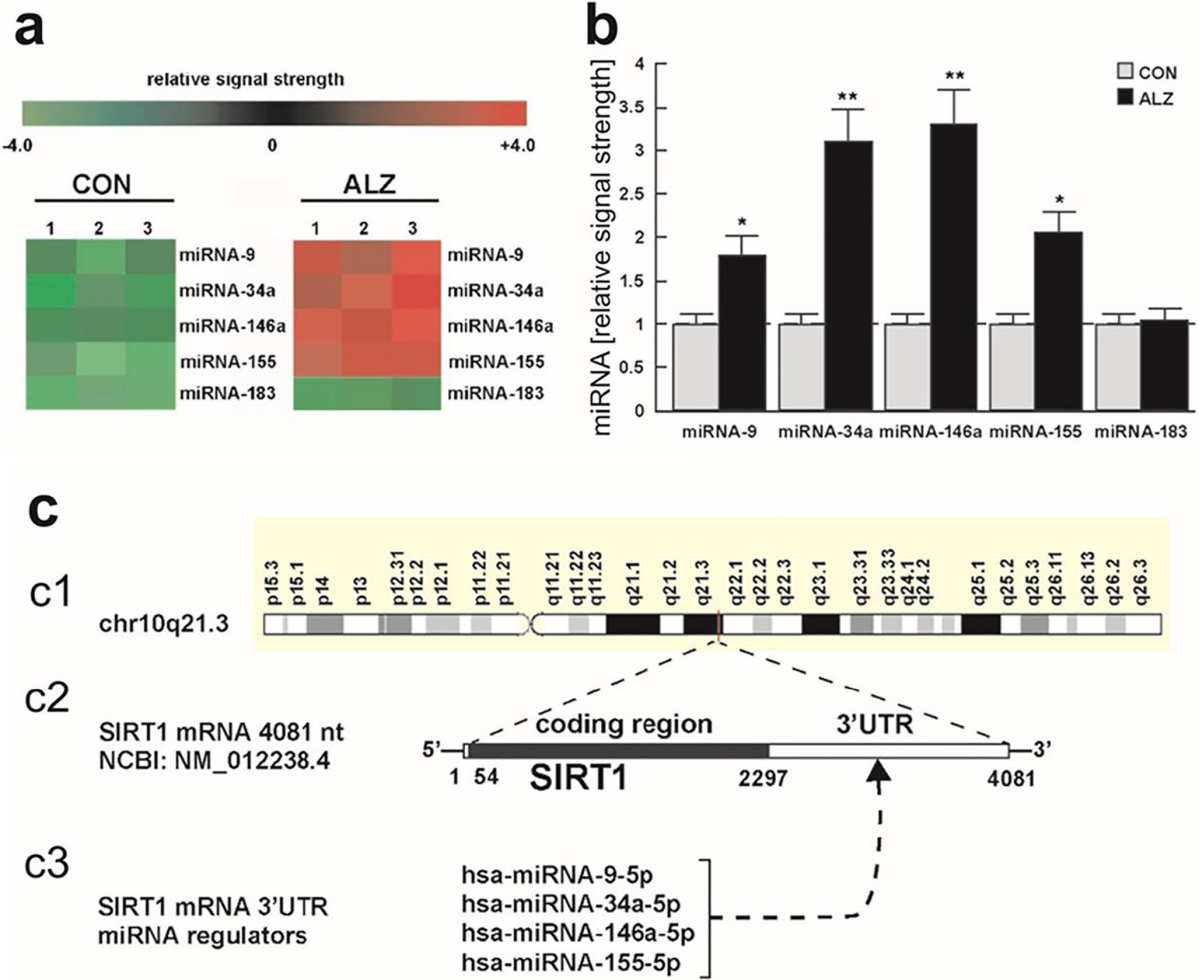
miRNA-upregulation and SIRT1 downregulation in AD brain. Brain samples from temporal cortex of AD brains and age-matched controls (all female) were used for isolation of miRNA. Analysis of miRNA was performed, as described above. The results of a miRNA-array based analysis of three controls versus three AD brains presented in cluster diagram ‘heat map’ format (**a**) and in bar graph format (**b**). Compared to age-, gender- and post-mortem interval-matched controls, miRNA-9, miRNA-34a, miRNA-146a and miRNA-155 are significantly upregulated in AD brain temporal neocortex to levels 1.7- to 3.3-fold above controls. **c** Part (c1) depicts chromosome 10q21.3 (chr10q21.3) from which a single copy SIRT1 mRNA is generated; part (c2) the SIRT1 mRNA is a 4081 nucleotide (nt) transcript consisting of a 54 nt 5′-leader sequence, a 2243 nt coding region and a 1784 nt 3′untranslated region (3′UTR); part (c3) interestingly the SIRT1 3’UTR contains recognition sequences for multiple miRNAs including miRNA-9, miRNA-34a, miRNA-146a and miRNA-155

Then, our further study on short post-mortem interval (PMI) human brain tissues using array-based miRNA-mRNA analysis and miRNA-mRNA-based linking assay showed a significant upregulation of miRNA-9, miRNA-34a, miRNA-146a and miRNA-155 in AD brain compared to age-, gender- and (PMI-matched) controls [49, 50]. Remarkably, each of these pro-inflammatory microRNAs has a binding site in the *SIRT1* mRNA 3′untranslated region (3’UTR) (Fig. 9).

## Discussion

It is widely accepted that Aβ-evoked oxidative stress plays crucial role in pathomechanism of AD. Moreover, it was also shown that microglia activation and mitochondria dysfunction may enhance production of reactive oxygen species (ROS) which subsequently could contribute to the progression of AD. In our study, we have investigated how Aβ oligomers influence expression of selected genes related to antioxidative defence and to mitochondrial function in microglial BV2 and in neuronal SH-SY5Y cells in culture. Our data indicated that AβO significantly affected viability of both types of cells; however, differently altered expression of genes encoding crucial antioxidative enzymes SOD1 and SOD2. The expression of gene for mitochondrial SOD2 (*Sod2*) was about seven times higher after 48 h of AβO treatment in BV2 cells, but the expression of gene for this enzyme in neuronal cells was significantly inhibited. However, *Sod1* transcription together with catalase (*Cat*) was enhanced by about 20–40% in neuronal SH-SY5Y cells. The significant differences were observed also in gene expression of sirtuins, as for example SIRT1 and SIRT3, which are involved in antioxidative defence [52]. In microglia BV2 cells, transcription of *Sirt1* and *Sirt3* was decreased, but in neuronal SH-SY5Y cells, only the gene expression for *Sirt5* was downregulated. These differences may play an important role in oxidative stress evoked by AβO in these two cells lines. It is also known that neurons are most vulnerable cells for oxidative injures, while microglia and astrocytes are much more resistant [53]. Oxidative damage is one of the earliest and the most important events in AD [54–58]. Aβ oligomers enhance free radical levels, and alter the balance between pro-oxidative and antioxidative processes leading to alterations of pro-survival kinases and several signalling pathways, neuronal impairment and synaptic degeneration which were indicated by many studies, including our recent data [15, 45, 46, 59].

Alteration of function of mitochondrial ETC complexes may be responsible for the enhancement of ROS production and energy alteration. The present study demonstrates that Aβ_1-42_ oligomers significantly affected the level of free radicals in neuronal cells SH-SY5Y but had no effect on the level of ROS in microglial BV2 cells. It seems that higher expression of SOD2 in BV2 cells may protect against free radicals’ accumulation and, in consequence, against their deleterious influences on macromolecules and mitochondrial function [15, 58]. However, until now, the role of AβO in modulation of gene expression related to oxidative stress and mitochondria function in microglia and in neuronal cells is not fully elucidated.

Our data indicated the lower expression of subunits of complex I and II of ETC in AβO-treated microglial BV2 cells. The significant alterations were observed after short-time incubation (24 h). These changes may indicate transient downregulation of microglial ETC function because after 48 h of AβO treatment, only gene expression for subunit of complex II (*Sdha*) was decreased. Activation of gene expression for *Sod2* in BV2 cells may suggest that it is a part of mechanism protecting mitochondria against ROS generated by impaired ETC in conditions of AβO toxicity. Analysis of gene expression of subunits of ETC in SH-SY5Y cells indicated lower level of mRNA for subunit of complex II (*Sdha*) after 24 h of AβO treatment and enhancement of subunit of complex I after 48 h. The activity of cytochrome c oxidase in BV2 cells was three times higher, comparing to activity of this enzyme in SH-SY5Y cells. Previous data showed that complex IV is affected in AD brain. It was demonstrated that cytochrome c oxidase (COX) activity is significantly decreased in the brains of AD patients, comparing to healthy controls [60, 61]. However, *in vitro* studies showed that the inhibitory effect of Aβ_1-42_ on COX activity is dependent on the presence of Cu^2+^ ions [62, 63]. This effect seems to be specific for copper ions, because it was not observed in the presence of Fe^2+^, Fe^3+^ or Zn^2+^. Copper-dependent inhibition of COX by Aβ_1-42_ requires reduced methionine-35 and seems to be dependent on the Aβ’s capacity to bind and reduce Cu^2+^.

The significantly higher *Sod2* expression and higher COX activity in microglial BV2 comparing to neuronal SH-SY5Y cells could be responsible for significant differences in maintaining mitochondria membrane potential (ΔΨm) in condition of AβO toxicity. Our results suggest that mitochondrial function and ATP production is preserved in BV2 cells, opposite to SH-SY5Y cells. Additionally, the significantly higher expression of *Bcl2* in BV2 comparing to SH-SY5Y cells may also have a positive effect on mitochondrial membrane integrity. ΔΨm in BV2 cells was not altered after 24 h of AβO treatment, comparing to control conditions. On the other hand, the level of free radicals was increased and ΔΨm was significantly decreased in SH-SY5Y cells by Aβ toxicity. The results indicate that AβO exerts toxic effect on SH-SY5Y cells, but in BV2 cells it activates several defensive mechanisms which could contribute to neuroprotective function of microglial cell in early stage of AβO toxicity.

It was previously demonstrated that SOD2 and other SODs play a critical role in inactivation of superoxide radicals and nitric oxide, preventing peroxynitrite formation and mitochondria dysfunction, which may lead to the extension of lifespan of flies and mice [64, 65]. Our results may suggest that SOD2 in microglia could be responsible for higher resistance of these cells to oxidative stress evoked by Aβ, comparing to neuronal cells. However, viability of both cells types is significantly affected by Aβ toxicity suggesting that different mechanisms could be responsible in regulation of these cells survival/death pathway. Activator of sirtuins, SRT1720, which particularly activates Sirt1, enhanced neuronal SH-SY5Y cell viability suppressed by AβO. However, in microglial cells, SRT1720 had no significant effect. In this study, inhibitor of poly(ADP-ribose)polymerase-1 (PARP-1) Olaparib exerted also cytoprotective action on neuronal cells under AβO toxicity and enhanced BV2 viability transiently after 24 h. These differences may be connected with significant downregulation of transcription of *Sirt1* and *Sirt3* in BV2 cells and probably with lower activity of these enzymes [23].

Sirtuins and poly(ADP-ribose)polymerases (PARPs) use β-NAD^+^ as a substrate, and it was proposed that inhibitors of PARPs are the most effective activators of Sirts [66]. The best characterized mammalian sirtuin is Sirt1. A lot of studies were carried out also on the role of Sirt3 in mitochondria, aging and neurodegeneration [23, 67]. These both Sirts control mitochondria function and antioxidative enzymes through deacetylation of several targets including SOD2, GPX, PGC1α, unfolding protein responses (UPR), FOXO signalling [23, 26, 27]. The NAD/sirtuin pathway influences lifespan, and it is suggested that activation of Sirts, with one exception of Sirt2, may protect the brain against neurodegeneration [27, 68–71]. However, a lot of questions in this field must be solved as indicated by Dang [72]. The data of Ekblad and Schuler [73] suggested that sirtuins are not affected by PARP inhibitors (including PJ34 and Olaparib). Our recently published data indicated that PARP inhibition enhanced significantly expression of *Sirt1* and *Sirt6* in the absence and in the presence of AβO and enhanced transcription of mitochondrial *Sirt4* [74].

In this study, activator of sirtuins (mainly Sirt1) SRT1720 and PARP1 inhibitor Olaparib considerably enhanced neuronal cell viability which was significantly decreased by AβO toxicity and had also protective effect on microglia viability after short time 24 h of AβO treatment. Our previous data showed that PARP1 inhibitor activated expression of several mitochondria-related genes in PC12 cells in control conditions and AβO toxicity. Concomitantly, AβO exerted significant time-dependent inhibitory effect on expression of genes encoding several proteins involved in regulation of mitochondrial dynamics and subunits of oxidative complexes [45].

Our research carried out on Tg-AD murine models indicated significant downregulation of gene transcription of *Sirt1*, which plays a crucial role in APP metabolism through the regulation of secretase alpha [27, 74]. Moreover, our study indicated the lower transcription of gene *Mnf1* and higher expression of gene encoding Drp1, the crucial protein for mitochondrial dynamics. Recent studies demonstrated abnormalities of mitochondria in AD evoked by altered homeostasis in transcription of genes for highly conserved proteins with GTP-ase activity which are responsible for changes of mitochondria fragmentation and fusion [21, 36, 44, 75, 76]. Manczak and colleagues indicated that partial reduction of Drp1 diminishes mitochondria dysfunction, maintains mitochondrial dynamics and enhances mitochondrial biogenesis and synaptic activity in APP^+^ mice. Decrease of the level of Drp1 may reduce production of Aβ and, in consequence, maintain axonal transport of mitochondria and supplying the sufficient quantities of ATP in AD neurons [44]. The expression and function of mitochondrial and synaptic proteins is significantly regulated by complex molecular processes including miRNA. It has been demonstrated that miRNA through post-transcriptional alteration may affect neuronal circuit development, maturation and function [77, 78]. Recent studies demonstrated a crucial role of miRNA in the pathomechanism of neurodevelopmental and neurodegenerative disorders (including AD). It is now well known that upregulated miRNAs predominantly act to decrease target mRNA levels *via* miRNA-mRNA-coupled signalling networks [49, 79]. Our finding demonstrated that brain-abundant miRNAs including miRNA-9, miRNA-34a, miRNA-146a and miRNA-155 are upregulated and target the *SIRT1* mRNA 3′UTR. These data may explain why *SIRT1* expression is found to be downregulated in AD. Anti-miRNA-based therapeutic strategies may be useful to normalize miRNA-9, miRNA-34a, miRNA-146a and/or miRNA-155 levels and restore homeostasis in AD or in brain pathologies where AβO is over-abundant.

## Conclusions

**Fig. 10:**
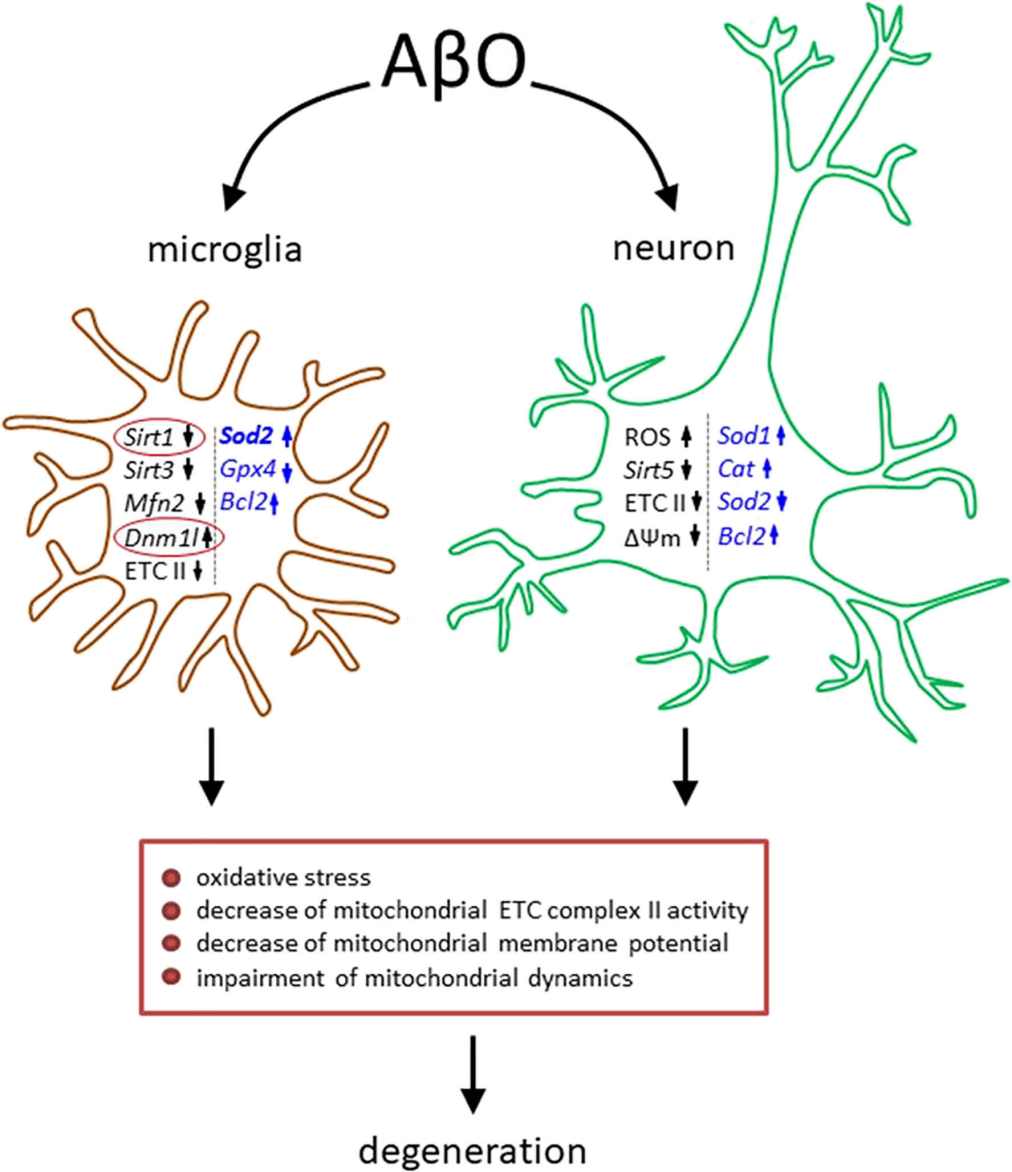
Schematic representation of main findings of the study

In summary, our study indicated the significant impact of Aβ oligomers on expression of several genes related to antioxidative defence and mitochondrial function and dynamics in neuronal and microglial cells and in the brain cortex of Tg-AD mice (Fig. 10). The most important finding of this study is downregulation of *Sirt1* gene in glia cells and in brain cortex of AD mice and high transcription of gene encoding DRP1, the key protein in mitochondria fragmentation. The overexpression of specific miRNAs in human AD brain may explain, in part, the downregulation of *SIRT1* that is crucial player in homeostatic mitochondrial operation, APP metabolism and the maintenance of redox state. On the basis of these data, we suggest that modulation of expression and/or activity of Mfn1, Drp1 and Sirt1, including anti-miRNA-based strategies, may be promising in the advancement of AD therapy.
